# The Treatment of Typhoid Fever

**Published:** 1905-04-15

**Authors:** 


### THE TREATMENT OF TYPHOID FEVER.

The treatment of typhoid fever is a subject the interest of which never fails. Instances of the disease in greater or less abundance fall to the lot of every practitioner, and, apart from the general responsibility of the case, there are possible emergencies which demand prompt judgment and resolute action. Hence, records of personal experiences including a number of carefully observed and accurately recorded cases are always welcome, and have the advantage of placing the lessons learned by the practitioner in attendance at the service of a large number of his brethren. Dr. H. E. Snell[1] has analysed a series of 100 cases occurring in the Coventry City Hospital, and his notes on treatment contain several interesting and useful features. First, on the question of diagnosis, it is noted that in the 100 cases 74 showed typical typhoid spots; in 19 the spleen was enlarged to the extent that it could be felt under the ribs; and in 26 of the cases Widal's test was applied and a positive result obtained with only one exception. There was but a single fatal case. Of drugs, Dr. Snell notes that while urotropin is credited with the ability to cause the typhoid bacilli to disappear from the urine, it has no favourable influence on the course of the disease. Turpentine in cases of hæmorrhage is of decided value and seems also to exercise a beneficial influence on the general condition of the patient; it may be given by the mouth or in an enema, and in one of the cases as much as half a drachm every hour was administered. In 57 of the 100 cases olive oil was given either by the stomach or per rectum, and

Dr. Snell considers with favourable results. The oil in half- to one-ounce doses may be ordered every four hours, and is best given floated on a little soda-water; in addition, a daily enema of 8 to 10 ozs. may be conveniently administered by Herschell's apparatus. Dr. Owen Paget, of Freemantle, has advocated very much larger doses of the oil, and describes its use as "a perfect boon to the general practitioner." It is suggested that olive oil acts partly as a food and partly as a sedative and emollient to the inflamed and ulcerated mucous membrane. Alcoholic stimulants, in the majority of Dr. Snell's cases, were not prescribed at all, but when ordered they were administered freely—in one case as much as 12 ozs. in the 24 hours. No antipyretic drugs were used, but tepid or cold sponging was employed whenever the temperature reached 104°, and this was sometimes supplemented by leaving the patient, after the sponging, covered only with a sheet. In reference to diet, Dr. Snell allowed his patients more milk than is usually permitted. Four to six pints was the daily allowance for an adult; this was given in small amounts frequently repeated, and if it did not agree with the patient it was peptonised, but continued in the same amount. Beef-tea was allowed when there was no diarrhœa. A return to solid food was sanctioned more readily than is the common practice. So soon as the temperature had been normal for two days, sopped bread-crumbs were permitted, and after a further few days boiled sole. Relapses were not more frequent than is the case with a more cautious dietary. Among the complications was one instance of an abdominal abscess of uncertain origin; it was opened and drained, and the patient made a good recovery.

Dr. J. Orton,[2] in a paper on the same subject, discussed among other questions the value of Widal's test. He pointed out that a negative result might be obtained any time before the tenth day of the disease, and that the same result might be observed in a very severe case in which the blood had not become immune. Hence a negative to Widal's test might mean either that the patient was not suffering from the disease, or that the test had been applied too early, or that the case was a severe one and the patient would probably die. On the other hand, a positive result means the patient either has or has had enteric fever, Osler having shown that the reaction may be present even 20 or 30 years after the attack. On the question of prognosis, Dr. Orton remarked that numerous sudamina which occurred irrespective of any tendency to perspiration were of good omen, even in a case where the chances seemed to be against the patient. For intestinal hæmorrhage turpentine internally, an ice compress to the abdomen, ice by the mouth, and strychnine hypodermically were recommended.

Dr. John Paton [3] records a case of intestinal perforation occurring in the course of enteric fever in a child of seven years and successfully treated by abdominal section. About the tenth day of the illness the child had a rigor and complained of abdominal pain. The pulse rose to 130 and the temperature to 104°; there was generalised abdominal tenderness and some rigidity of the muscles of the lower abdomen. On the following day there was constant vomiting, tympanitic distension of the abdomen, and obliteration of the liver dulness, with the general signs of collapse. The abdomen was therefore opened and a small perforation was found about a foot from the lower end of the ileum. In spite of an attack of acute lobar pneumonia which developed 11 days after the operation, the patient made a complete recovery. Perforation is a rare event in the enteric fever of children, and the case bears out the general view that the chance of an operation is better in early life than in later years.

[1] Brit. Med. Jour., Feb. 25, 1905, p. 414. [2] Ibid., p. 413. [3] Ibid., p. 413.

